# The grades and freshness assessment of eggs based on density detection using machine vision and weighing sensor

**DOI:** 10.1038/s41598-021-96140-x

**Published:** 2021-08-17

**Authors:** Supakorn Harnsoongnoen, Nuananong Jaroensuk

**Affiliations:** The Biomimicry for Sustainable Agriculture, Health, Environment and Energy Research Unit, Department of Physics, Faculty of Science, Mahasarakham University, Kantarawichai, 44150 Mahasarakham Thailand

**Keywords:** Electrical and electronic engineering, Techniques and instrumentation

## Abstract

The water displacement and flotation are two of the most accurate and rapid methods for grading and assessing freshness of agricultural products based on density determination. However, these techniques are still not suitable for use in agricultural inspections of products such as eggs that absorb water which can be considered intrusive or destructive and can affect the result of measurements. Here we present a novel proposal for a method of non-destructive, non-invasive, low cost, simple and real—time monitoring of the grading and freshness assessment of eggs based on density detection using machine vision and a weighing sensor. This is the first proposal that divides egg freshness into intervals through density measurements. The machine vision system was developed for the measurement of external physical characteristics (length and breadth) of eggs for evaluating their volume. The weighing system was developed for the measurement of the weight of the egg. Egg weight and volume were used to calculate density for grading and egg freshness assessment. The proposed system could measure the weight, volume and density with an accuracy of 99.88%, 98.26% and 99.02%, respectively. The results showed that the weight and freshness of eggs stored at room temperature decreased with storage time. The relationship between density and percentage of freshness was linear for the all sizes of eggs, the coefficient of determination (R^2^) of 0.9982, 0.9999, 0.9996, 0.9996 and 0.9994 for classified egg size classified 0, 1, 2, 3 and 4, respectively. This study shows that egg freshness can be determined through density without using water to test for water displacement or egg flotation which has future potential as a measuring system important for the poultry industry.

## Introduction

Freshness plays an important role in determining the quality of eggs and egg products^1^. It is the most relevant index used in the food industries, transportation and consumers use it to assess nutritional value and food products. Methods for measuring the freshness of eggs can be divided into two approaches: destructive and non-destructive measurement methods^2^. Destructive measurement methods are traditional, time-consuming biochemical methods (sensory indicators, Haugh unit, pH value, Yolk index and total volatile basic nitrogen). However, samples tested in this way cannot be reused. Therefore, only a few samples can be tested. There are several main advantages of non-destructive measurement of egg freshness, such as fast detection speed, no-pretreatment need for stripping eggshell and suitability for on-line detection^3^. As a result of the above advantages, nondestructive methods of egg freshness measurement have gained widespread attention.

Many non-destructive testing techniques have been proposed for the freshness assessment of eggs; such as assessments based on visible transmission spectroscopy^4,5^, near infrared (NIR) spectroscopy^6–9^, visible-near infrared (VIS–NIR) spectroscopy^10–14^, Fourier transform near infrared (FT-NIR) spectroscopy^15^, Raman spectroscopy^16,17^, dielectric properties^18,19^, electronic nose (E-nose)^20–27^, hyperspectral images^28–30^, ultrasound^31^, and machine vision^32–35^. Egg freshness testing techniques based on spectroscopy (visible, NIR, VIS–NIR, FT-NIR and Raman) are fast, efficient and nondestructive approaches, but they cannot evaluate the internal quality of eggs directly and might be affected by the eggshell (color and thickness of the eggshell) or environmental factors (temperature, humidity and stability of illuminating light). Evaluating egg freshness by their dielectric properties is another interesting non-destructive testing technique. However, there are still problems with the variability in egg shapes and mass. As well as the duplication of measurements that cannot be guaranteed. Egg freshness testing techniques based on an electronic nose provide a rapid and non-destructive method, but it is not sensitive enough to acquire the smell information produced by different degrees of freshness from an intact egg. Testing egg freshness using hyperspectral imaging is another non-destructive technique. However, such techniques require a fast computer, sensitive detector and large storage capabilities for analyzing hyperspectral data. Egg freshness testing techniques based on the ultrasound are classified as nonthermal treatments. Nevertheless, this technique produces free radicals, which can negatively impact and damage the product quality due to oxidation and there are difficulties selecting the appropriate parameters to obtain the accuracy and precision in detection. Nowadays, machine vision is one method that can be used to detect egg freshness that has many advantages such as fast, online estimation and non-destructive methodology. Egg freshness determination using machine vision proposed by Cai et al., had the advantage is that it can be measured dynamically^32^. However, this method also has errors caused by mechanical vibration during measurement. In addition, the system did not evaluate egg grading alongside the freshness measurement. Qinghua et al. proposed the detection of cracked eggs by machine vision which is a simple and intelligent system^33^ but this study also did not analyze the freshness and grade of eggs in the measurement system. Egg freshness determination using machine vision was proposed by Wang et al.^34^ and Soltani et al.^35^. The advantages of both proposed methods are a simple system that can measure freshness in a variety of ways. However, both proposed systems lacked a study of the classification of eggs by the system. We perceive that non-destructive measurements of egg freshness and grade discrimination in the same system based on real-time weight and density measurement principles have not been proposed before. This technique is simple and effective. It can also solve the problem of egg freshness measurement in water displacement and flotation that is not suitable for practical monitoring of products because the eggs absorb water; and this can be considered intrusive or destructive and can affect the test^35^. In order to easily recognize the quality and freshness of eggs at a lower cost, non-destructive, non-invasive and real—time monitoring is desirable. We therefore present the design, validation and development of the grade and freshness assessment of eggs by weight and density using load cell detection and machine vision techniques in this work.

## Materials and methods

### Egg samples

The eggs used for the test were 107 fresh eggs from the farm of Mahasarakham University, Mahasarakham province, Thailand. The samples were divided into two subsets consisting of a calibration set and a model set. Calibration sets were used to proposed system calibration, while a model set was used to create mathematical models and test the accuracy of the models. The calibration set contained 20 samples, the remaining 87 samples constituted the model set. The model sets contained 87 samples in which the eggs were divided into 5 groups based on weight and size, size classification number 0, jumbo (70–100 g), size classification number 1, extra-large (65–69.99 g), size classification number 2, large (60–64.99 g), size classification number 3, medium (55–59.99 g) and size classification number 4, small (50–54.99 g).

### Density measurement system and calibration

It is well known that the egg freshness can be measured by the floatation method in which eggs are placed in a glass or container of water which is a simple initial test of egg freshness^36^. But this method requires a long time to prepare the equipment and to test it. It is also inappropriate for testing large numbers of eggs in a short time. In addition, it was observed that the relationship between density and the principle egg freshness from such methods has not been clearly studied and modeled. The relationship between density and egg freshness are investigated in this work. In addition, egg freshness is determined using the float method, which is a destructive measurement and can affect the measurement results. Because the eggs absorb water during the measurement process, this problem can be solved by the techniques presented. The density formula is as follows:

1 $$ \rho = \frac{m}{V} $$

where ρ is density, m is mass and V is volume.

**Figure 1:**
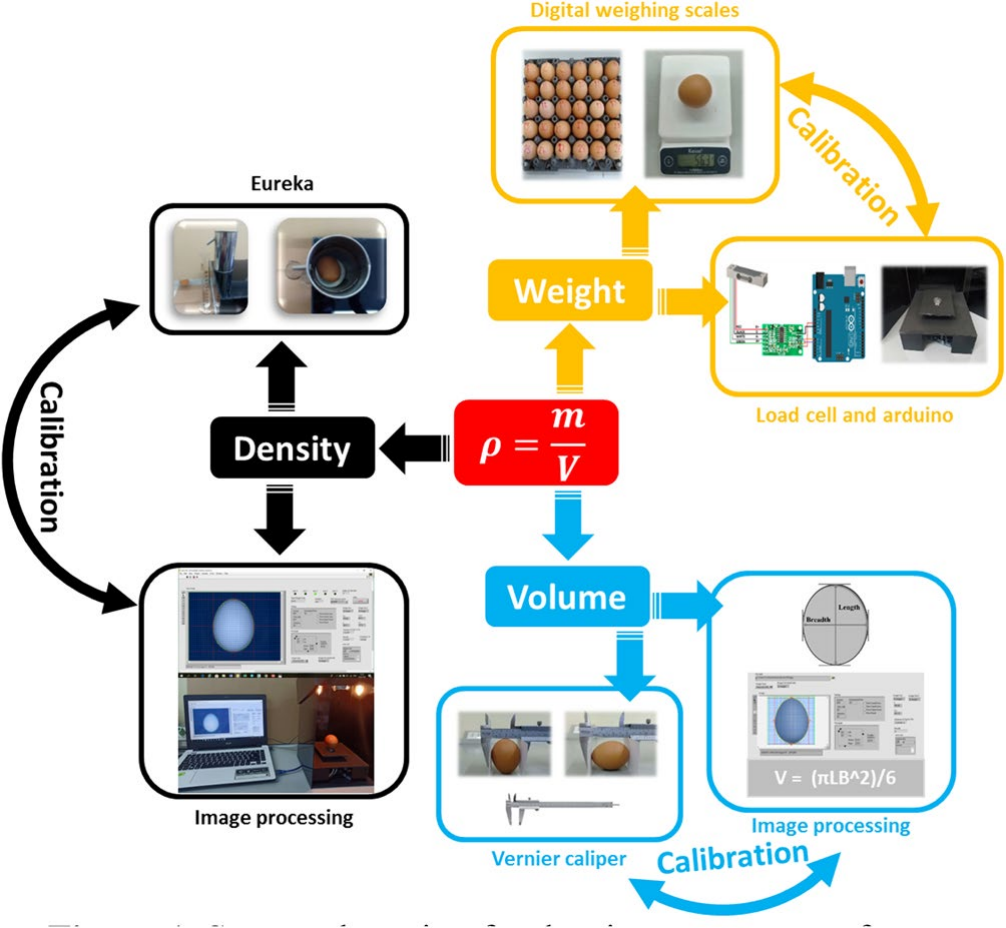
System detection for density assessment of eggs.

**Figure 2:**
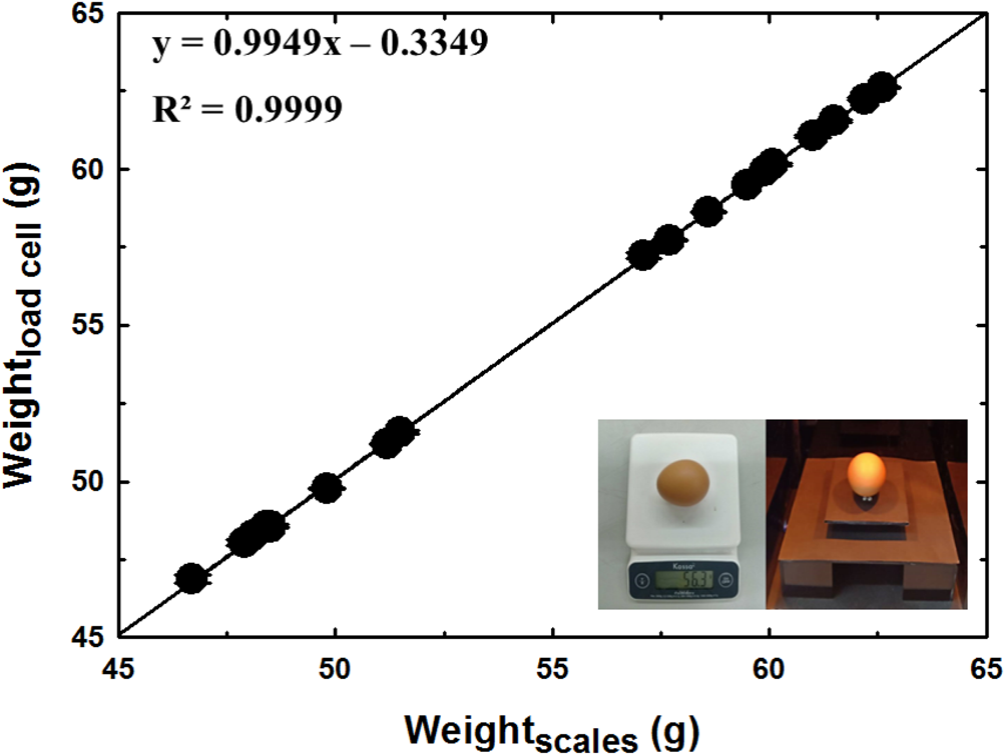
Measurement of weight and calibration.

The density measurement system and calibration are shown in Fig. 1. The measurement system was divided into three parts. The first part was the weight determination of load cell and was calibrated with a digital weighing scale. The second part was the volume determination by machine vision (length, breadth and volume) and calibrated by Vernier caliper. The third part was the density determination, the weight from the first part and the volume of the second part were used to calculate egg density based on Eq. (1) and calibrated by Eureka can. The total parameters were measured, analyzed, recorded and displayed by computer as shown in Fig. 2. The schematic display on the computer screen consisted of picture, weight, length, breadth, volume, density, size classification number and percentage of egg freshness.

#### Weight measurement

The 20 eggs samples came directly from the chicken farm of Mahasarakham University and were divided into 4 groups based on weight of eggs, size classification number 1 (8 egg), size classification number 2 (2 egg), size classification number 3 (6 egg) and size classification number 4 (4 egg). On the first day, the weight, length, breadth and volume of 20 samples were measured using standard instruments and the proposed method. A load cell sensor was used to detect the weight of the eggs in this system; it was interfaced with as an Arduino board for converting analog signals to digital signals. The digital signals were recorded and analyzed by personal computer. The accuracy of the proposed system was checked by a digital weighing scale. The results obtained by the digital weighing scale and the load cell sensor are compared in Fig. 2. It was determined that both measurement techniques had a reasonable level of agreement with the averaged relative error being 0.12% (accuracy was 99.88%) and the maximum relative error being 0.33% in linear measurements (accuracy was 99.67%). The slope of the regression value for the linear regression of egg weight was 0.9949 and the coefficient of determination (R^2^) value was 0.9999. A possible contributory source of this measurement error might be difference load cell assembly and load cell resolution. However, the level of error is small and considered to be acceptable.

#### External physical characteristic measurement

**Figure 3:**
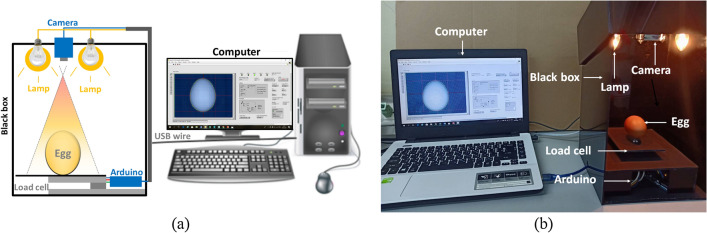
The proposed system (**a**) design system and (**b**) build system.

Image processing techniques used to measure the length and width of the eggs are illustrated in Fig. 3a, while Fig. 3b shows the actual generation system. The system was divided into two parts. The first part was the hardware and the second part was the control program. The system’s measuring components were housed in a black box, lamp, camera, load cell and Arduino board. The creation of a uniform light around the sample was important and required carefully design. Accordingly, in order to provide suitable illumination and to eliminate the effects of environmental noises, a black rectangular box of 20 × 20 × 30 cm was fabricated. A 15 W LED lamp was installed at the top of the box. A camera was used and fixed approximately 20 cm above the egg sample. The camera had 680 × 480 pixels in horizontal and vertical directions, respectively. The camera was attached to a personal computer through a LabVIEW program.

**Figure 4:**
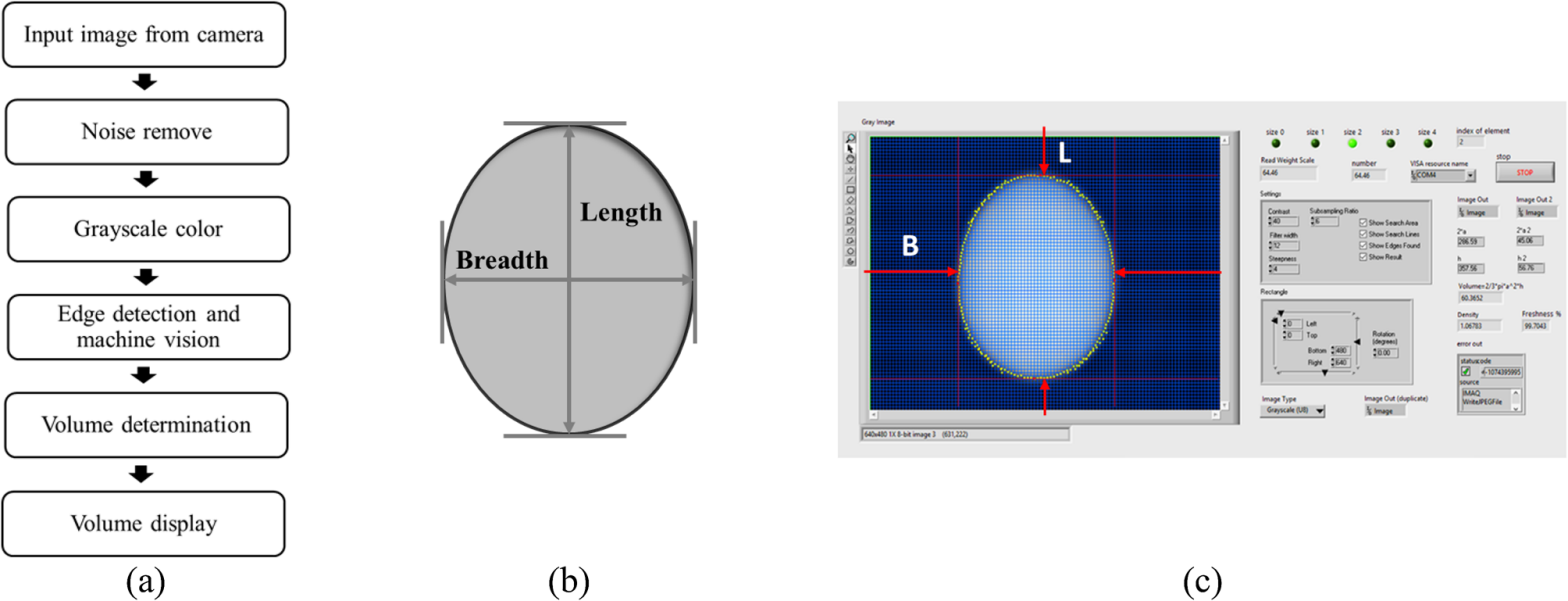
Egg parameters for calculate SI and V (**a**) image processing step, (**b**) measured parameters and (**c**) calculated volume and display parameters.

The image processing system that was used in this study is shown in the block diagram in Fig. 4a. In the first step, the camera detects image sample. In the second step, the noise was removed from the input image. In the third step, the image color was converted to grayscale. In the fourth step, the edge image of eggs was detected and the length (L) and breadth (B) of eggs was determined as shown in Fig. 4b. In the fifth step, the length and breadth of eggs were used to calculate volume and displayed as is shown in Fig. 4c. The length and breadth of the egg were determined using an egg shape measurer and their SI^37,38^:

2 $$ SI = \left( \frac{W}{L} \right) \times 100\% $$

The mathematic model for calculating the volume of an egg based on image processing is as follows^39^:

3 $$ V = \frac{{\pi LB^{2} }}{6} $$

where *V* is the volume (mm^3^), length is L (mm), and the maximum breadth is B (mm).

**Figure 5:**
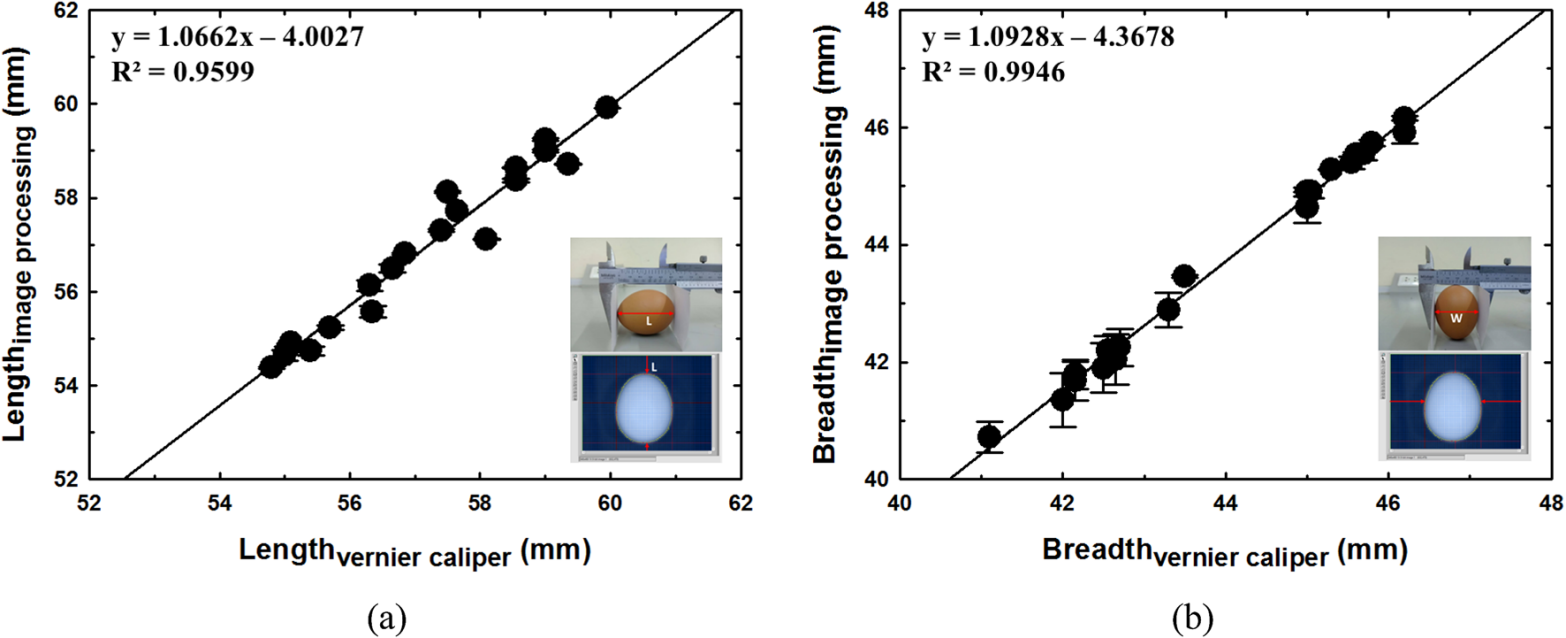
Measurement of external physical characteristic and calibration (**a**) length and (**b**) breadth.

The length and breadth are used to calculate the shape index (SI) and volume of eggs. A Vernier caliper was used to check the accuracy of the proposed method. The results obtained by the image processing and Vernier caliper are compared in Fig. 5. It was determined that the length and breadth of both measurement techniques had a reasonable level of agreement with the averaged relative error being 0.57% (accuracy was 99.43%) and 0.67% (accuracy was 99.33%), respectively. While the maximum relative error being 1.70% (accuracy was 98.30%) and 1.55% (accuracy was 98.45%) in linear measurements, respectively. The slopes of the linear regression obtained from the relationship between length and width were 1.0662 and 1.0928, with determination coefficients (R^2^) of 0.9599 and 0.9946, respectively. There are several possible sources which might contribute to the measurement errors. The first two are the instrumental error and observation error. The third may be from the perspective effect along the optical path of the camera which could cause small variations of the length and breadth conversion coefficients across an image of the egg. However, the level of error is small and considered acceptable.

**Figure 6:**
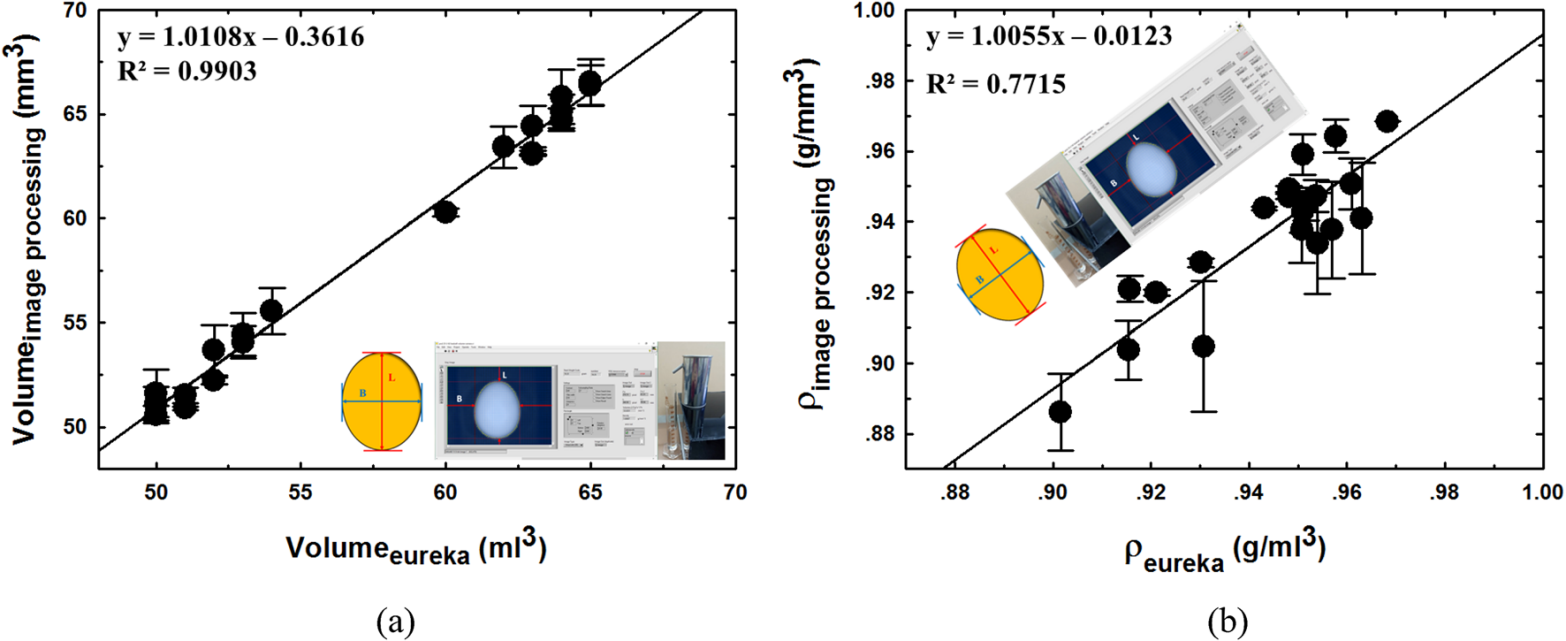
Measurement of volume and density of egg (**a**) volume and (**b**) density.

The volume and density determination based on Archimedes' principle and the imaging processing are compared in Fig. 6a and b, respectively. It was found that the volume and density determined by both measurement techniques had a reasonable level of agreement with the averaged relative error being 1.74% (accuracy was 98.26%) and 0.98% (accuracy was 99.02%), respectively. While the maximum relative error was 3.25% (accuracy was 96.75%) and 2.80% (accuracy was 97.20%) in linear measurements. The slopes of the linear regression obtained from the relationship between length and breadth were 1.0108 and 0.9638 with the coefficient of determination (R^2^) value being 1.0055 and 0.7715, respectively. There are several possible sources of measurement errors. The first two are instrumental error and observation error. The third may be from the perspective effect along the optical path of the camera which could cause small variations of the length and breadth conversion coefficients across an image of the egg. The fourth may be due to cumulative errors caused by weight measurements and physical features that have occurred in previous processes. The fifth may be due to an error caused by the density determination based on the Archimedes' principle measurement process. However, the level of error was small and considered acceptable.

The average relative error, the maximum relative error, accuracy, slope, R^2^, RMSEC (root mean square error calibration) and RPD (residual prediction deviation) of weight and external physical characteristic measurement are shown in Table 1.

**Table 1:** The relative error, accuracy, statistical indicator and relationship of weight and external physical characteristic measurement of eggs.

Measurement	Averaged relative error (%)	Average accuracy (%)	Maximum relative error (%)	Minimum accuracy (%)	Slope	R^2^	RMSEC	RPD
Weight	0.12	99.88	0.33	99.67	0.9949	0.9999	0.0488	118.5642
Length	0.57	99.43	1.70	98.30	1.0662	0.9599	0.34605	5.1209
Breadth	0.67	99.33	1.55	98.45	1.0928	0.9946	0.1320	13.9197
Volume	1.74	98.26	3.25	96.75	1.0108	0.9903	0.6002	10.4388
Density	0.98	99.02	2.80	97.20	1.0055	0.7715	0.0097	2.1462

## Results and discussion

### Weight as function of storage time

**Figure 7:**
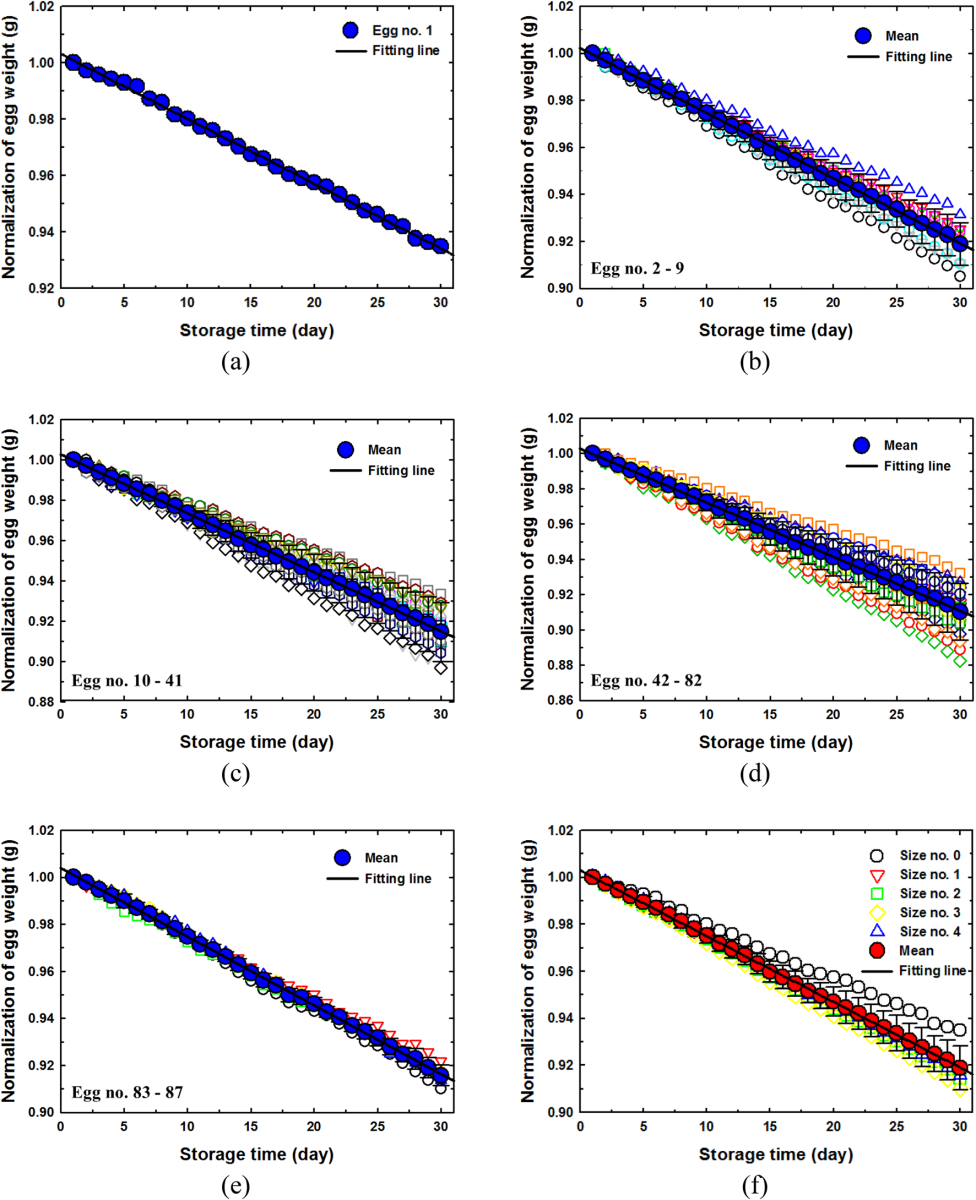
Weight as function on storage time (**a**) size classification number 0, (**b**) size classification number 1, (**c**) size classification number 2 (**d**) size classification number 3, (**e**) size classification number 4 and (**f**) all size classification numbers.

The 87 eggs were divided into 5 groups; size classification number 0, 1 egg (weight 76.60 g); size classification number 1, 8 eggs (average weight 66.79); size classification number 2, 32 eggs (average weight 62.33 g); size classification number 3, 41 eggs (average weight 57.75 g) and size classification number 4, 5 eggs (average weight 53.65 g). The weight, length, breadth and volume of the 87 eggs of the model set were measured daily for 30 days. Figure 7a–e shows the relationship between storage time and normalized weight of egg, and the linear regression and average linear regression between weight of eggs in the each size. The results clearly demonstrate that the weight of eggs in all size classes decreased linearly with the storage time. The experiment results clearly showed that eggs that were kept at room temperature for 30 days experienced a reduction in egg weight of 0.0023–0.0031 times the initial weight. The average linear regression between weight and storage time of eggs in the each size classification number and R^2^ values were calculated, as shown in Table 2. The slope of the regression values for the size classification numbers 0, 1, 2, 3 and 4 are −0.0023, −0.0028, −0.0029, −0.0031 and −0.0028 and the R^2^ values were 0.9982, 0.9995, 0.9996, 0.9996 and 0.9994 for validation, respectively.

**Table 2:** Linear regression equation between weight of size classification numbers 0, 1, 2, 3 and 4 with storage time.

Size classification number	Regression equation	R^2^
0	Y = −0.0023 X + 1.0030	0.9982
1	Y = −0.0028 X + 1.0023	0.9995
2	Y = −0.0029 X + 1.0027	0.9996
3	Y = −0.0031 X + 1.0028	0.9996
4	Y = −0.0029 X + 1.0039	0.9994
Average	Y = −0.0028 X + 1.0030	0.9996

Figure 7f shows average linear regression of egg in all sizes. The linear regression equation and the R^2^ of all size classification numbers are shown in Table 2, where Y is normalizing of weight and *x* is time (day). The results showed that egg size, classification number 3 had the highest rate of weight loss. This may be because it had physical properties that allowed water to evaporate more easily than other egg sizes. Egg size classification number 0 have the lowest weight loss rate compared to other egg sizes. However, the exact cause of this issue has not been proven conclusively in this study, and requires future study.

### The relationship between weight and density

**Figure 8:**
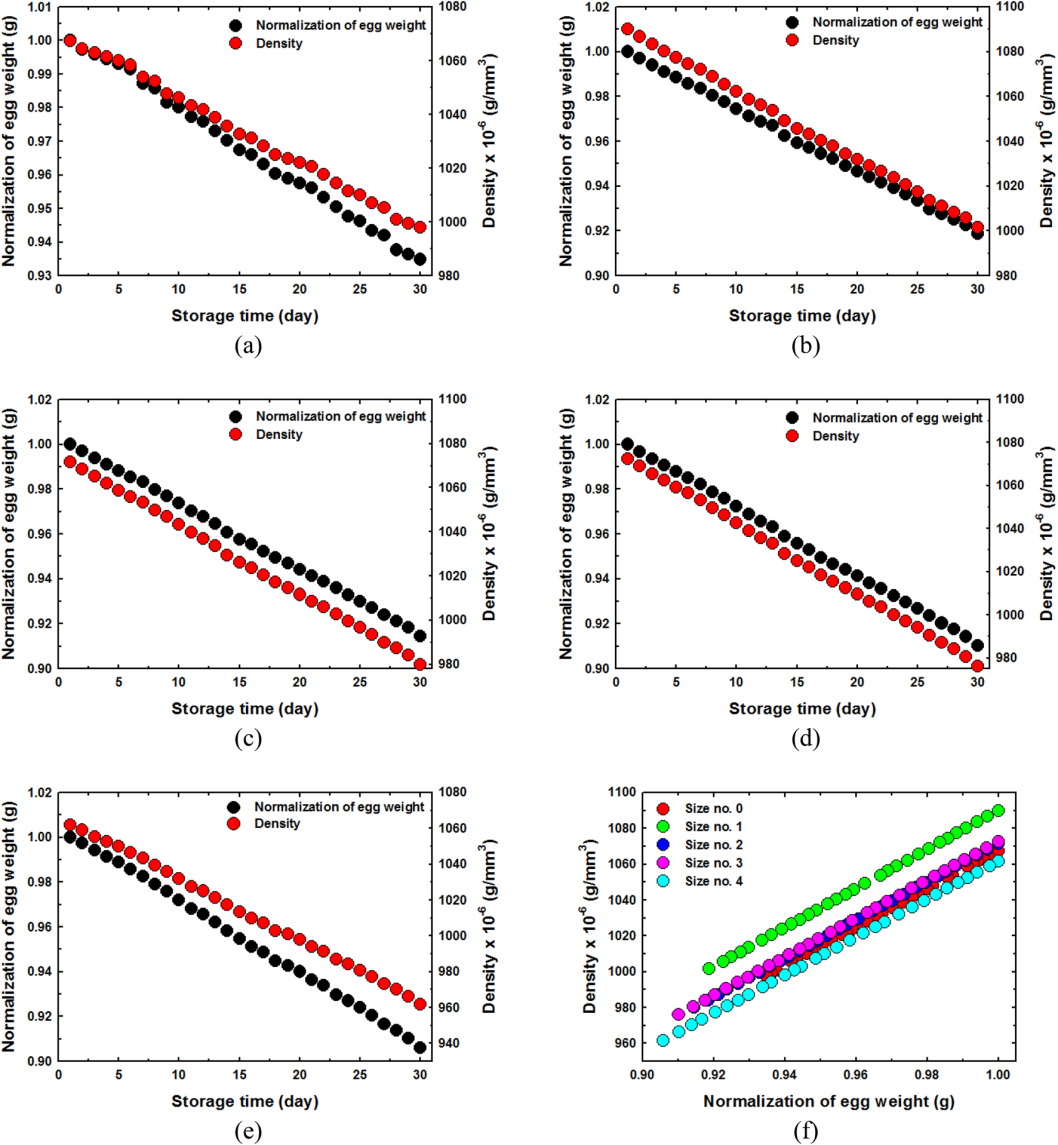
Weight and density as function of storage time (**a**) size classification number 0, (**b**) size classification number 1, (**c**) size classification number 2 (**d**) size classification number 3, (**e**) size classification number 4 and (**f**) relationship between normalized weight and density of all eggs sizes.

**Figure 9:**
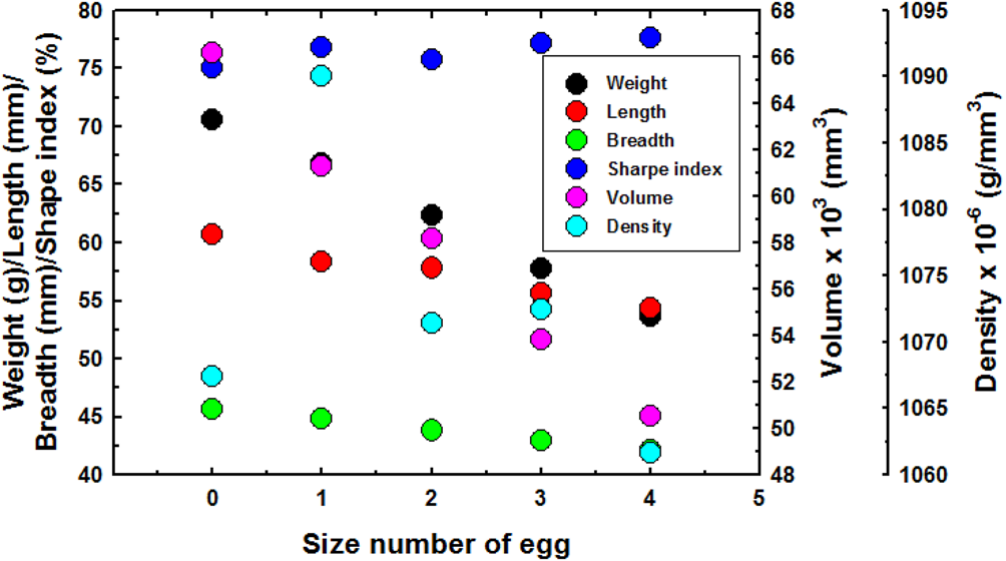
Relationship between egg size with the means of weight, length, breadth, shape index, volume and density of the eggs at the time of maximum freshness.

Figure 8a–e show the relationship between weight and density with the storage time for egg size, classification numbers 0 to 4. It is clear that the weight and density varies directly with the storage time. The weight and density of eggs decreased with increased storage time. Egg weight and density decreased during storage due to the loss of water evaporating through thousands of pores in the eggshell surface^40^. Over time, the spongy epidermises of the eggshell begin to dry and shrink. This causes the pores of the eggshell to increase in size and number and his makes it easier for gas and moisture to escape from the egg. As a result, the weight of the eggs decreased with longer storage periods. The environment (temperature and humidity) plays a key role in the loss of moisture from the eggs by evaporation. Carbon dioxide is lost through the pores of the shell while oxygen enters the egg and creates bubbles inside instead of moisture and carbon dioxide. As a result of this, the egg weight and density are reduced^41^. The relationship between normalized weight and density of eggs of all sizes was linear, as shown in Fig. 8f. The relationship between egg size and the means of weight, length, breadth, shape index, volume and density of eggs at the time of maximum freshness is shown in Fig. 9. The data on egg size and physical properties are shown in Table 3. The results clearly show that weight, length, breadth and volume decreased with increased egg size. On the other hand, it was found that the shape index increased with increasing egg size. This is because the length of the egg has a higher rate of reduction than the width as the egg size increases.

**Table 3:** The egg size and physical properties of the eggs examined at the highest egg freshness.

Size	Mean weight (g)	Mean L (mm)	Mean B (mm)	Shape index (%)	Mean V (mm^3^)	Density, ρ × 10^–6^ (g/mm^3^)
0	70.60	60.75	45.60	75.06	66,141.58	1067.41
1	66.79	58.29	44.80	76.86	61,275.20	1090.00
2	62.33	57.82	43.83	75.80	58,172.48	1071.47
3	57.75	55.68	42.98	77.19	53,846.41	1072.50
4	53.65	54.33	42.15	77.58	50,535.45	1061.63

The results clearly showed that the eggs of each size class had different moisture loss rates and initial freshness. The results showed that eggs of size classification number 1 had the highest initial density, but had a narrow weight and density range. Eggs of size classification number 4 had the lowest initial density, but had a wide weight and density range. Egg size classification number 0 has the narrowest range of weight and density, while the rate of moisture loss and primary freshness of egg size, classification numbers 0, 2, 3 and 4 were similar.

### The relationship between freshness and density

**Figure 10:**
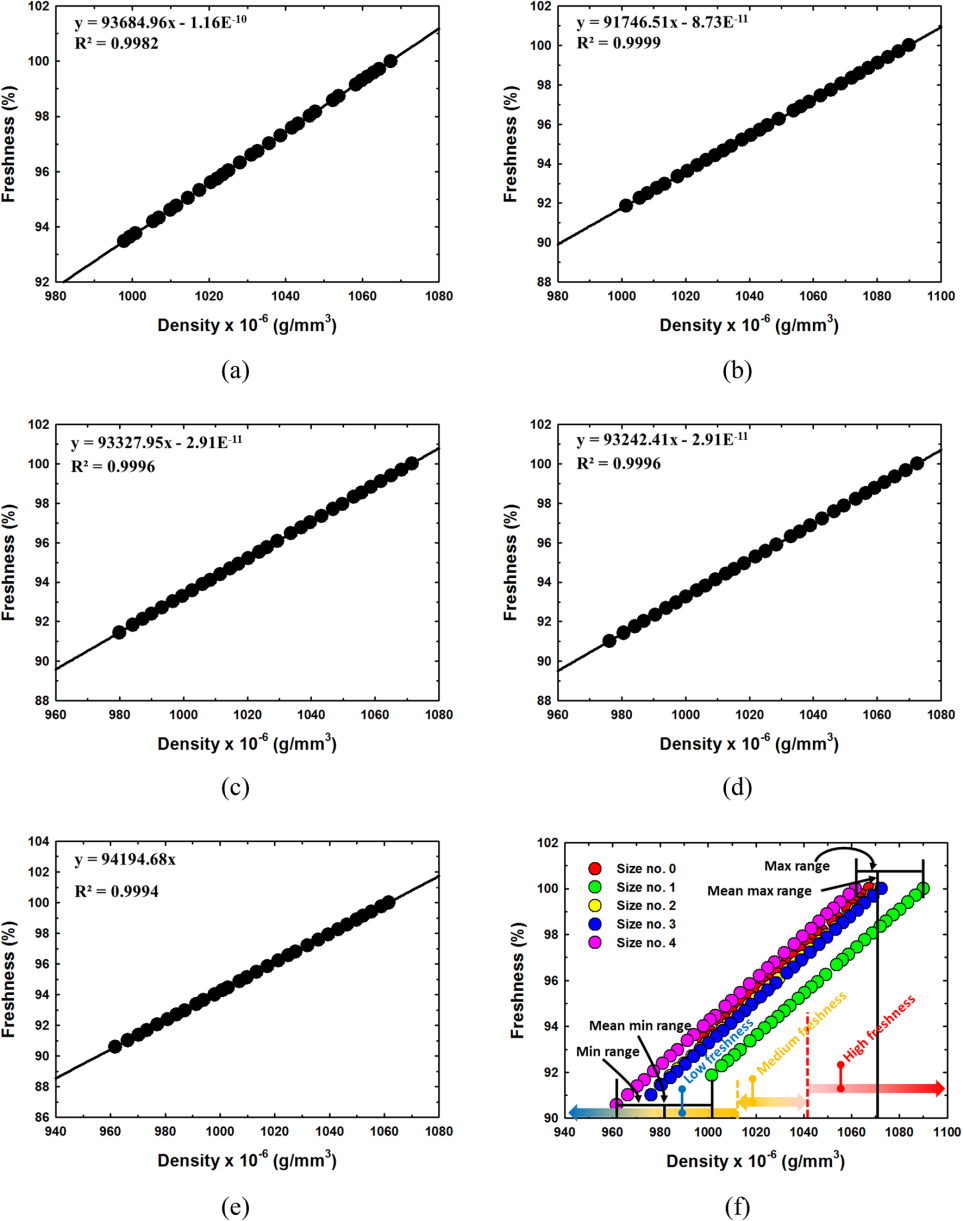
Freshness as function of density (**a**) size classification number 0, (**b**) size classification number 1, (**c**) size classification number 2 (**d**) size classification number 3, (**e**) size classification number 4 and (**f**) the range of freshness.

The relationships between density and percentage of egg freshness to the storage time of all egg sizes are shown in Fig. 10a–e. The results clearly showed that the egg density and percentage of freshness were linearly related, the R^2^ values were as large as 0.9982, 0.9999, 0.9996, 0.9996 and 0.9994 for egg size, classification numbers 0, 1, 2, 3 and 4, respectively. We developed and presented the Egg Freshness Equation in percentage form in this study, as shown in Eq. (4).

$$ Freshness\mathop {}\limits^{{}} (\% ) = \frac{{\rho_{E} }}{{\rho_{HE} }} \times 100\mathop {}\limits^{{}} \% $$

where $\rho_{{\text{E}}}$ is the density of the eggs tested after they were stored at different times.

$\rho_{{{\text{HE}}}}$ is the initial density (the highest freshness) of each egg size.

The results showed that the densities of eggs of each size at first laying had a different freshness as shown in Fig. 10f. We averaged the initial densities for each egg size using the sensed changed freshness of eggs in each egg size class as shown in Table 3. It is clear that freshness and density were linearly related in all egg sizes. The results clearly showed that the maximum densities of all egg sizes were in the range of 1061.63 × 10^–6^ (g/mm^3^) – 1090.00 × 10^–6^ (g/mm^3^) and the mean of maximum density was 1072.60 × 10^–6^ (g/mm^3^). The minimum densities of all egg size were in the range of 961.70 × 10^–6^ (g/mm^3^) – 1001.40 × 10^–6^ (g/mm^3^) and the mean of maximum density was 983.41 × 10^–6^ (g/mm^3^). The difference between the mean of maximum density and mean of minimum density was 98.19 × 10^–6^ (g/mm^3^). We would like to divide the range of freshness based on density into three grades, high, medium and low freshness as shown in Fig. 10f. Therefore, the difference of mean maximum and minimum divided by three is 29.73 × 10^–6^ (g/mm^3^). This value was then used as a criterion for dividing egg freshness into 3 ranges for grading: high freshness (> 1042.87 × 10^–6^ (g/mm^3^), medium freshness (1013.14 × 10^–6^ (g/mm^3^) – 1042.87 × 10^–6^ (g/mm^3^)) and low freshness (< 1013.14 × 10^–6^ (g/mm^3^)).

### Comparison with other machine vision system

A comparison of grades and freshness assessment of eggs in this work and using other machine vision methods is reported in Table 4. Machine vision reported in Ref.^32^ and Ref.^34^ was proposed for determination of egg freshness through the dynamic weighing, area ratio of egg yolk, area ratio of air room and height ratio of air room, which is presented egg freshness sensing same as proposed in this work, but it is not proposed graded method and clearly divided freshness range. The sensing of cracked eggs is based on length, breadth and LFI index as proposed in Ref.^33^, but freshness was not tested graded of eggs was not reported. Ref.^35^ proposed egg quality sensing through diameter and volume detection, which is presented as egg freshness sensing based on volume detection. This approach was the same as proposed in this work, but did not propose a grading method and clearly dividing the freshness range based on density and weight.

**Table 4:** The comparison of difference sensor for egg freshness detection based on machine vision.

Refs.	System	Monitoring	Equation	Sensing data
^32^	Machine vision and dynamic weighing system	Freshness	$HU = \alpha + \beta /R_{L} = \alpha + \beta \frac{{M_{f} }}{{M_{d} }}$	Dynamic weighing (*M*_*d*_)
^33^	Machine vision system	Cracks	NA	Length, breadth and LFI index
^34^	Machine vision system	Freshness	$y_{1} = - 0.0094x + 1.0549$ $y_{2} = - 0.5517e^{ - 0.0359x}$ $y_{3} = - 0.0037x + 0.3989$	Area ratio of egg yolk, area ratio of air room and height ratio of air room
^35^	Dielectric properties, machine vision, and artificial neural network (ANN) techniques	Haugh unit, yolk index, yolk/albumen, yolk weight	$HU = 100\log \left[ {HA - 5.67\left( {\frac{{30M^{0.37} - 100}}{100}} \right) + 1.9} \right]$ $YolkIndex = \frac{Yolkheigh(mm)}{{Yolkdiameter(mm)}}$ $Yolk/Albumen = - \frac{YolkWeight}{{AlbumenWeight}} \times 100$	Diameter and volume
Present work	Machine vision and weighing system	Graded and freshness	$Freshness(\% ) = \frac{{\rho_{E} }}{{\rho_{HE} }} \times 100\%$	Weight, length, breadth, volume and density

*NA* data not available, *HU* Haugh uni, *α* *and* *β* regression coefficients, *M*_*f*_ weight of fresh egg estimated by long and minor axis sizes, *M*_*d*_ the weight measured by dynamic weighing, *y*_*1*_ the area ratio of egg yolk, *y*_*2*_ the area ratio of air room, *y*_*3*_ the height ratio of air room, *HA* the height of the thick

Albumen, *M *the egg mass (g).

## Conclusions

A freshness assessment and grading of eggs by weight and density are presented in this work. Egg grading is classified into five grades by using weight as a sorting criterion. The freshness of eggs was divided into 3 periods: high, medium and low freshness, using density as the criterion. The proposed system can be used effectively for non-destructive, non-invasive detection that is low cost, low weight, small size, flexible and easy to operate. Moreover, it can be record data, analyze data, monitor physical characteristics and classify egg freshness in real time. This proposed measuring system is potentially important for the poultry industry and biological research in the future.

## References

[1] Romdhane K, Bart K, Flip B, Bart DK, Eddy D, Josse DB (2006). Methods to evaluate egg freshness in research and industry: a review. Eur. Food Res. Technol..

[2] Dejian D, Tao J, Wei L, Xuan S, Rui X, Jingwei Z (2020). Nondestructive detection for egg freshnessbased on hyperspectral scattering image combined with ensemble learning. Sensors.

[3] Liang Q, Mao-cheng Z, Zhong L, De-hong S, Jun L (2020). Non-destructive testing technology for raw eggs freshness: a review. SN Appl. Sci..

[4] Kemps B, Bamelis F, De Ketelaere B, Mertens K, Tona K, Decuypere E, De Baerdemaeker JD (2006). Visible transmission spectroscopy for the assessment of egg freshness. J. Sci. Food Agric..

[5] Liu Y, Ying Y, Ouyang A, Li Y (2007). Measurement of internal quality in chicken eggs using visible transmittance spectroscopy technology. Food Control.

[6] Coronel-Reyes J, Ramirez-Morales I, Fernandez-Blanco E, Rivero D, Pazos A (2018). Determination of egg storage time at room temperature using a low-cost NIR spectrometer and machine learning techniques. Comput Electron Agric.

[7] Zhao J, Lin H, Chen Q, Huang X, Sun Z, Zhou F (2010). Identification of egg’s freshness using NIR and support vector data description. J. Food Eng..

[8] Jie DF, Wang XJ, Wei X (2016). Research on the detection model of egg freshness based on the near-infrared spectroscopy technology. Food Mach..

[9] Lin H, Zhao JW, Sun L, Chen QS, Zhou F (2011). Freshness measurement of eggs using near infrared (NIR) spectroscopy and multivariate data analysis. Innov. Food Sci. Emerg. Technol..

[10] Abdanan, M. S., Minaei, S., Hancock, N.H., Karimi, Torshizi, M. A. An intelligent system for egg quality classifcation based on visible-infrared transmittance spectroscopy. *Inf. Process Agric.* **1**, 105–114 (2014).

[11] Aboonajmi M, Saberi A, Abbasian NT, Kondo N (2016). Quality assessment of poultry egg based on visible–near infrared spectroscopy and radial basis function networks. Int. J. Food Prop..

[12] Duan YF, Wang QH, Ma MH, Lu X, Wang CY (2016). Study on non-destructive detection method for egg freshness based on LLE-SVR and visible/near-infrared spectrum. Guang Pu Xue Yu Guang Pu Fen Xi/Spectrosc Spectr Anal.

[13] Dong XG, Dong J, Li YL, Xu HB, Tang XY (2019). Maintaining the predictive abilities of egg freshness models on new variety based on VIS–NIR spectroscopy technique. Comput Electron Agric.

[14] Dong XG, Dong J, Peng YK, Tang XY (2017). Comparative study of albumen pH and whole egg pH for the evaluation of egg freshness. Spectrosc Lett.

[15] Giunchi A, Berardinelli A, Ragni L, Fabbri A, Silaghi FA (2008). Nondestructive freshness assessment of shell eggs using FT-NIR spectroscopy. J. Food Eng..

[16] Liu Y, Ren X, Yu H, Cheng Y, Guo Y, Yao W, Xie Y (2020). Non-destructive and online egg freshness assessment from the egg shell based on Roman spectroscopy. Food Control.

[17] Joshi R, Lohumi S, Joshi R, Kim MS, Qin J, Baek I, Cho B (2019). Raman spectral analysis for non-invasive detection of external and internal parameters of fake eggs. Sens. Actuators B Chem..

[18] Lau S, Subbiah J (2018). An automatic system for measuring dielectric properties of foods: Albumen, yolk, and shell of fresh eggs. J. Food Eng..

[19] Sun J, Liu B, Mao H, Wu X, Gao H, Yang N (2016). Non-destructive examination for freshness of eggs based on dielectric properties and yolk index regression model. Trans. Chin. Soc. Agric. Eng..

[20] Xiang X, Wang Y, Yu Z, Ma M, Zhu Z, Jin Y (2019). Non-destructive characterization of egg odor and fertilization status by SPME/GC-MS coupled with electronic nose. J. Sci. Food Agric..

[21] Yimenu S, Kim J, Kim B (2017). Prediction of egg freshness during storage using electronic nose. Poult. Sci..

[22] Liu M, Pan LQ, Tu K, Liu P (2010). Determination of egg freshness during shelf life with electronic nose. Nongye Gongcheng Xuebao/Trans Chin Soc Agric Eng.

[23] Li JT, Wang J, Li Y, Wei Y (2017). Detection of egg freshness using electronic nose. Modern Food Sci Technol.

[24] Li JT, Zhu SS, Jiang S, Wang J (2017). Prediction of egg storage time and yolk index based on electronic nose combined with chemometric methods. LWT Food Sci Technol.

[25] Liu P, Tu K (2012). Prediction of TVB-N content in eggs based on electronic nose. Food Control.

[26] Deng FF, Chen W, Wang J, Wei ZB (2018). Fabrication of a sensor array based on quartz crystal microbalance and the application in egg shelf life evaluation. Sens Actuators B Chem.

[27] Yongwei W, Wang J, Zhou B, Lu Q (2009). Monitoring storage time and quality attribute of egg based on electronic nose. Anal. Chim. Acta.

[28] Suktanarak S, Teerachaichayut S (2017). Non-destructive quality assessment of hens’ eggs using hyperspectral images. J. Food Eng..

[29] Siche R, Vejarano R, Aredo V, Velasquez L, Saldaña E, Quevedo R (2015). Evaluation of food quality and safety with hyperspectral imaging (HSI). Food Eng. Rev..

[30] Zhang W, Pan L, Tu S, Zhan G, Tu K (2015). Non-destructive internal quality assessment of eggs using a synthesis of hyperspectral imaging and multivariate analysis. J. Food Eng..

[31] Aboonajmi M, Setarehdan SK, Akram A, Nishizu T, Kondo N (2014). Prediction of poultry egg freshness using ultrasound. Int. J. Food Prop..

[32] Sun L, Yuan L, Cai J, Lin H, Zhao J (2014). Egg freshness on-line estimation using machine vision and dynamic weighing. Food Anal. Methods.

[33] Guanjun B, Mimi J, Yi X, Shibo C, Qinghua Y (2019). Cracked egg recognition based on machine vision. Comput. Electron. Agric..

[34] Qiaohua W, Xiaoyan D, YiLin R, Youchun D, Lirong X, Zhou P, Youxian W, Shucai W (2009). Egg freshness detection based on digital image technology. Sci. Res. Essays.

[35] Soltani M, Omid M, Alimardani R (2015). Egg quality prediction using dielectric and visual properties based on artifcial neural network. Food Anal Methods.

[36] Lisa AS, Mary KC (2006). Density determination by water displacement and flotation: an introductory experiment in forensic chemistry. J. Chem. Educ..

[37] Anderson KE, Tharrington JB, Curtis PA, Jones FT (2004). Shell characteristics of eggs from historic strains of single comb white leghorn chickens and relationship of egg shape to shell strength. Int. J. Poult. Sci..

[38] Reddy PM, Reddy VR, Reddy CV, Rap SP (1979). Egg weight, shape index and hatchability in khaki Campbell duck egg. Ind. J. Poult. Sci..

[39] Narushin VG, Lu G, Cugley J, Romanov MN, Griffin DK (2020). A 2-D imaging-assisted geometrical transformation method for non-destructive evaluation of the volume and surface area of avian eggs. Food Control.

[40] Jozefa K, Sokolowicz Z (2015). Effect of chicken breed and storage conditions of eggs on their quality. Acta Scientiarum. Polonorum. Zootechnica.

[41] Hassan A, Aylin AO (2009). Effect of storage time, temperature and hen age on egg quality in free range layer. J Anim Vet Adv.

